# The prognostic value of interaction between mean corpuscular volume and red cell distribution width in mortality in chronic kidney disease

**DOI:** 10.1038/s41598-018-19881-2

**Published:** 2018-08-08

**Authors:** Chew-Teng Kor, Yao-Peng Hsieh, Chia-Chu Chang, Ping-Fang Chiu

**Affiliations:** 1 Division of Nephrology, Department of Internal Medicine, Changhua Christian Hospital, Changhua, Taiwan; 2 School of Medicine, Kaohsiung Medical University, Kaohsiung, Taiwan; 3 School of Medicine, Chung Shan Medical University, Taichung, Taiwan; 4 Ph.D. program in translational medicine, College of Life Science, National Chung Hsing University, Taichung, Taiwan

## Abstract

Recently, both red cell distribution width (RDW) and mean corpuscular volume (MCV) have been associated with unfavorable outcomes in several medical conditions. Therefore, we conducted this retrospective study of 1075 patients with stage 3–5 chronic kidney disease to investigate whether interactions between RDW and MCV influence the risk of mortality. These patients were divided into four groups: group A (n = 415), RDW ≤ 14.9% and MCV ≤ 91.6 fL; group B (n = 232), RDW > 14.9% and MCV ≤ 91.6 fL; group C (n = 307), RDW ≤ 14.9% and MCV > 91.6 fL; and group D (n = 121), RDW > 14.9% and MCV > 91.6 fL. The adjusted hazard ratio (HR) of all-cause mortality for group B versus group A was 1.44 (95% confidence interval [CI], 1.14–2.12, p = 0.02), group C versus group A 2.14 (95% CI, 1.31–3.48, p = 0.002), and group D versus group A 5.06 (95% CI, 3.06–8.37, p < 0.001). There was a multiplicative interaction between MCV and RDW in predicting patient mortality. The use of RDW in conjunction with MCV may improve healthcare by identifying those at an increased risk for mortality compared with the use of either RDW or MCV alone.

## Introduction

Anemia is a common condition encountered in many clinical settings, and is associated with adverse clinical outcomes. Mean corpuscular volume (MCV) is the measure of the average volume of circulatory red blood cells. Based on the value of MCV, anemia can be classified as microcytic, normocytic or macrocytic, which can aid in making a differential diagnosis. Normocytic anemia is the most common form in patients with chronic kidney disease (CKD)^1^. Red cell distribution width (RDW) is an index of erythrocyte size heterogeneity, and the combination of RDW and MCV has been shown to aid in identifying the cause of anemia.

In addition to its role in anemia, there has been a great increase in the amount of research investigating the role of RDW as an independent and significant predictor for mortality in many medical conditions in the last decade. For example, RDW has been demonstrated to be associated with unfavorable outcomes in heart failure, coronary artery disease, acute pulmonary embolism, septic shock, acute cerebral infarction, acute kidney injury treated with continuous renal replacement therapy and end-stage renal disease^2–10^. MCV has also been reported to be associated with adverse outcomes, however only a few studies have reported on the association between MCV and mortality. The majority of these studies have focused on cancer patients; however MCV has also been reported to be a predictor for mortality in patients undergoing primary coronary interventions with acute decompensated heart failure and CKD^11–14^.

Both RDW and MCV have therefore emerged as novel risk factors for negative outcomes, and both are now routinely reported as part of a complete blood cell count. We were interested in whether the concurrent use of these two hematological parameters can improve the prediction of patient mortality, and thus we conducted this study to investigate whether interactions between RDW and MCV influence the risk of mortality in patients with stage 3–5 CKD.

## Results

### Baseline characteristics of the study cohort

Of the 1075 participants, 470 (43.72%) were women. The mean age of the whole study cohort was 64.2 ± 12.35 years with group D being the oldest, and the mean BMI was 25.17 ± 4.15 kg/m^2^. The mean duration of follow-up was 2.35 ± 1.65 years, and the three leading underlying etiologies of CKD were diabetes mellitus (39.35%), hypertension (23.81%) and chronic glomerulonephritis (10.23%). Seven hundred and ninety eight patients (74.23%) were not current smokers, and 905 (84.19%) were never drinkers. The relevant information of the four groups is shown in Table 1. Group A had the highest BMI (25.69 ± 4.18 kg/m^2^) while group D had the lowest BMI (24.72 ± 3.98 kg/m^2^). Concerning the prevalence of medical comorbidities, there were significant differences in malignancy, congestive heart failure, diabetes mellitus, hyperlipidemia and liver cirrhosis among the four groups. However, there were no significant differences in the prevalence of cerebrovascular disease, chronic lung disease, coronary artery disease, dementia, hypertension and peripheral artery disease. With regards to the laboratory parameters, there were significant differences in all measurements among the four groups except for cholesterol level. Regarding the medications use, there were significant differences in the prescriptions for iron preparations, folic acid supplements, and erythropoiesis stimulating agents among the four groups.

**Table 1:** Baseline characteristics of the study population by MCV and RDW.

Variable	Group A n = 415	Group B n = 232	Group C n = 307	Group D n = 121	p-value
	RDW ≤ 14.9% MCV ≤ 91.6 fL	RDW > 14.9% MCV ≤ 91.6 fL	RDW ≤ 14.9% MCV > 91.6 fL	RDW > 14.9% MCV > 91.6 fL	
**Sex**					0.014^*^
Female	158(48.02%)	101(48.33%)	147(37.4%)	64(44.44%)	
**Age** (years)	59.07 ± 13.47	63.39 ± 12.17	67.1 ± 10.76	69.23 ± 9.32	<0.001^*^
**BMI** (kg/m^2^)	25.69 ± 4.18	25.12 ± 4.15	24.91 ± 4.16	24.72 ± 3.98	0.037^*^
**Smoker**					0.094
Non-current	259(78.72%)	152(72.73%)	278(70.74%)	109(75.69%)	
Current	70(21.28%)	57(27.27%)	115(29.26%)	35(24.31%)	
**Drinker**					0.167
Never	283(86.02%)	183(87.56%)	315(80.15%)	124(86.11%)	
Current	21(6.38%)	11(5.26%)	31(7.89%)	6(4.17%)	
Former	25(7.6%)	15(7.18%)	47(11.96%)	14(9.72%)	
**Comorbidity**					
Cancer	18(5.47%)	14(6.7%)	42(10.69%)	28(19.44%)	<0.001^*^
Cerebrovascular disease	53(16.11%)	34(16.27%)	50(12.72%)	22(15.28%)	0.537
Chronic lung disease	36(10.94%)	38(18.18%)	63(16.03%)	21(14.58%)	0.099
Congestive heart failure	36(10.94%)	34(16.27%)	29(7.38%)	23(15.97%)	0.003^*^
Coronary artery disease	72(21.88%)	60(28.71%)	94(23.92%)	43(29.86%)	0.152
Dementia	6(1.82%)	3(1.44%)	14(3.56%)	6(4.17%)	0.21
Diabetes mellitus	183(55.62%)	122(58.37%)	159(40.46%)	67(46.53%)	<0.001^*^
Hyperlipidemia	148(44.98%)	84(40.19%)	138(35.11%)	51(35.42%)	0.04^*^
Hypertension	249(75.68%)	156(74.64%)	285(72.52%)	92(63.89%)	0.057
Liver cirrhosis	2(0.61%)	2(0.96%)	6(1.53%)	7(4.86%)	0.006^*^
Peripheral artery disease	6(1.82%)	2(0.96%)	6(1.53%)	1(0.69%)	0.731
**Medication prescription**					
ACE inhibitor/ARB	226(68.69%)	147(70.33%)	241(61.32%)	90(62.5%)	0.064
Vitamin B12 (cyanocobalamin)	57(17.33%)	37(17.7%)	93(23.66%)	37(25.69%)	0.053
Iron preparations	44(13.37%)	62(29.67%)	38(9.67%)	25(17.36%)	<0.001^*^
Folic acid	43(13.07%)	50(23.92%)	81(20.61%)	40(27.78%)	0.001^*^
Erythropoiesis stimulating agents	56(17.02%)	59(28.23%)	58(14.76%)	36(25%)	<0.001^*^
Vitamin D	9(2.74%)	4(1.91%)	11(2.8%)	7(4.86%)	0.432
**Laboratory data**					
Albumin (g/dL)	3.79 ± 0.6	3.38 ± 0.72	3.82 ± 0.62	3.57 ± 0.62	<0.001^*^
BUN (mg/dL)	46.02 ± 23.6	52.61 ± 23.77	41.01 ± 20.35	50.89 ± 24.89	<0.001^*^
Creatinine (mg/dL)	3.58 ± 2.34	3.91 ± 2.44	3.24 ± 2.08	3.76 ± 2.18	0.003^*^
eGFR (ml/min per 1.73 m^2^)	23.07 ± 13.23	20.58 ± 12.13	24.63 ± 12.63	20.72 ± 11.68	<0.001^*^
Ca (mg/dL)	8.82 ± 0.68	8.61 ± 0.67	8.85 ± 0.59	8.81 ± 0.85	<0.001^*^
Phosphorus (mg/dL)	4.27 ± 1.09	4.45 ± 1.24	4.01 ± 1.05	4.16 ± 1.03	<0.001^*^
Cholesterol (mg/dL)	191.25 ± 48.9	183.7 ± 66.21	182.77 ± 47.74	179.94 ± 58.92	0.089
Triglyceride (mg/dL)	169.92 ± 115.9	161.37 ± 123.95	140.04 ± 82.97	121.64 ± 74.94	<0.001^*^
GPT (U/L)	19.71 ± 16.84	22.65 ± 18.26	25.4 ± 39.57	28.74 ± 29.76	0.007^*^
WBC count (/μL)	7624.49 ± 2369.9	8000.21 ± 2658.42	7129.99 ± 2398.77	7436.34 ± 2746.14	<0.001^*^
Hemoglobin (g/dL)	10.96 ± 2.04	9.57 ± 1.96	11.09 ± 2.12	10.21 ± 2.11	<0.001^*^
Uric acid (mg/dL)	8.17 ± 1.83	8.2 ± 1.88	7.68 ± 1.73	8 ± 1.86	0.001^*^
24- hour proteinuria (mg)	1242.9 ± 1967.1	1715.2 ± 2298.2	2044.4 ± 2795.7	2501.9 ± 3201.5	<0.001^*^

Values are expressed as mean ± SD or number (percentage).

CKD, chronic kidney disease; eGFR, estimated glomerular filtration rate; BMI, body mass index;

ACE inhibitor, angiotensin-converting enzyme inhibitor; ARB, angiotensin II receptor blocker.

*p < 0.05.

### The interaction between MCV and RDW in predicting all-cause mortality

**Figure 1:**
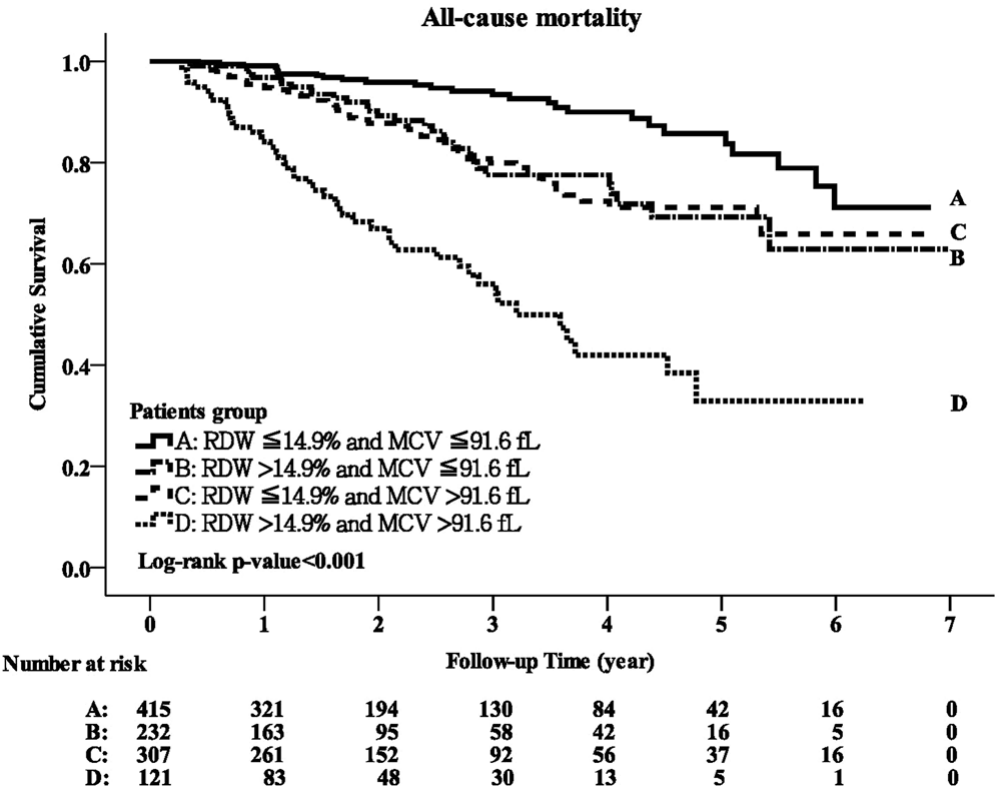
Kaplan-Meier curve of overall patient survival according to the groups stratified by MCV and RDW (log-rank test, p < 0.001).

Overall, 158 patients died during a mean follow-up period of 2.35 ± 1.65 years, including 28 (6.75%) in group A, 31 (13.36%) in group B, 51 (16.61%) in group C and 48 (39.67%) in group D. There was a significant difference in the crude mortality rate for the four groups (p < 0.001), and also a significant difference in Kaplan-Meier survival curves (log-rank test, p < 0.001; Fig. 1). Table 2 lists the unadjusted and adjusted HRs of Cox proportional hazard analysis for the outcomes among the four groups. There was a stepwise increase in the risk of all-cause mortality from group A to group D in the unadjusted and adjusted models (both p for trend <0.001). The adjusted HR for group B versus group A was 1.44 (95% CI, 1.14–2.12, p = 0.02), group C versus group A 2.14 (95% CI, 1.31–3.48, p = 0.002), and group D versus group A 5.06 (95% CI, 3.06–8.37, p < 0.001). The adjusted HR for group D was higher than the expected value determined by the addition of adjusted HRs for group B and C, suggesting a multiplicative interaction between MCV and RDW in predicting overall mortality for patients with stage 3–5 CKD.

**Table 2:** Cox proportional hazard models of clinical outcomes for the groups stratified by RDW and MCV.

Group	All-cause mortality				CVD mortality				Infection related mortality			
	crude HR (95% CI)	p-value	adjusted HR (95% CI)	p-value	crude HR (95% CI)	p-value	adjusted HR (95% CI)	p-value	crude HR (95% CI)	p-value	adjusted HR (95% CI)	p-value
A: RDW ≤14.9% and MCV ≤91.6 fL	1.000	—	1.000	—	1.000	—	1.000	—	1.000	—	1.000	—
B: RDW >14.9% and MCV ≤91.6 fL	2.25(1.35,3.75)	0.002*	1.44(1.14,2.12)	0.02*	0.98(0.25,3.93)	0.989	0.51(0.11,2.25)	0.37	3.56(1.69,7.49)	0.001*	2.15(0.95,4.88)	0.06
C: RDW ≤14.9% and MCV >91.6 fL	2.36(1.49,3.75)	<0.001*	2.14(1.31,3.48)	0.002*	2.40(0.89,6.50)	0.08	2.62(0.87,7.88)	0.08	3.08(1.52,6.23)	0.002*	2.96(1.41,6.22)	0.004*
D: RDW >14.9% and MCV >91.6 fL	7.26(4.54,11.59)	<0.001*	5.06(3.06,8.37)	<0.001*	9.98(3.87,25.75)	<0.001*	10.76(3.48,33.21)	<0.001*	7.56(3.59,15.93)	<0.001*	4.7(2.12,10.4)	<0.001*
^†^p-value for trend	<0.001		<0.001		<0.001		<0.001		<0.001		<0.001	

^†^A test for trend was conducted by treating group A to D as a continuous variable.

*p < 0.05.

### The interaction between MCV and RDW in predicting CVD-related mortality

**Figure 2:**
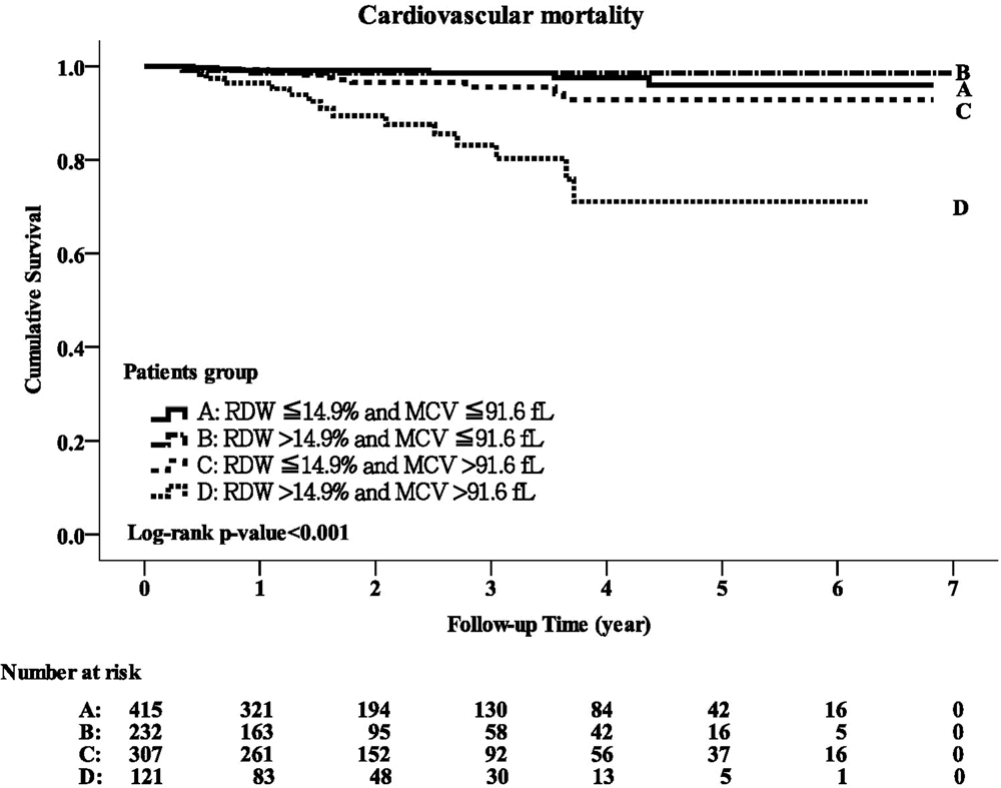
Kaplan-Meier curve of cardiovascular disease-related survival according to the groups stratified by MCV and RDW (log-rank test, p < 0.001).

Overall, 35 patients died of CVD during a mean follow-up period of 2.35 ± 1.65 years, including six (1.25%) in group A, three (1.23%) in group B, 11 (3.58%) in group C and 15 (12.4%) in group D. There was a significant difference in the crude CVD-related mortality rate for the four groups (p < 0.001), and also a significant difference in Kaplan-Meier survival curves (log-rank test, p < 0.001; Fig. 2). As shown in Table 2, there was a stepwise increase in the risk of CVD-related mortality from group A to group D in the unadjusted and adjusted models (both p for trend <0.001). The adjusted HR for group B versus group A was 0.5 (95% CI, 0.11–2.25, p = 0.37), group C versus group A 2.62 (95% CI, 0.87–7.88, p = 0.08), and group D versus group A 10.76 (95% CI, 3.48–33.21, p < 0.001).

### The interaction between MCV and RDW in predicting infection-related mortality

**Figure 3:**
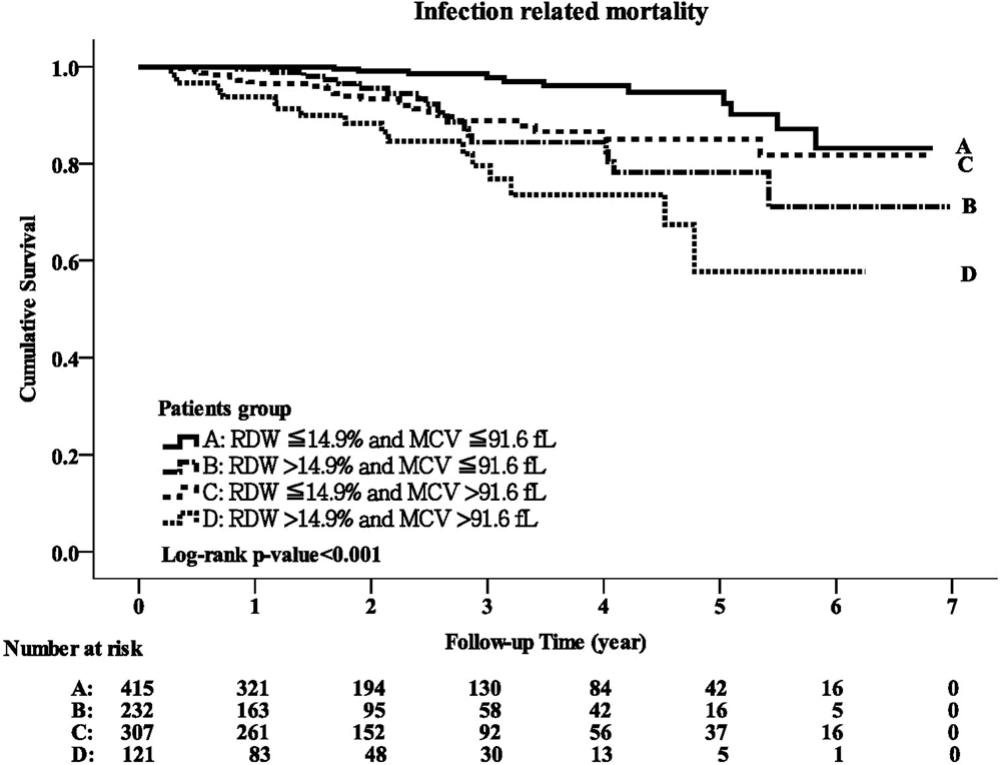
Kaplan-Meier curve of infection-related survival according to the groups stratified by MCV and RDW (log-rank test, p < 0.001).

Overall, 75 patients died of infection during a mean follow-up period of 2.35 ± 1.65 years, including 11 (2.65%) in group A, 19 (8.19%) in group B, 26 (8.47%) in group C and 19 (15.7%) in group D. There was a significant difference in the crude infection-related mortality rate for the four groups (p < 0.001), and also a significant difference in Kaplan-Meier survival curves (log-rank test, p < 0.001; Fig. 3). There was a stepwise increase in the risk of infection-related mortality from group A to group D in the unadjusted and adjusted models of Cox regression analyses (both p for trend <0.001; Table 2). The adjusted HR for group B versus group A was 2.15 (95% CI, 0.95–4.88, p = 0.06), group C versus group A 2.96 (95% CI, 1.41–6.22, p = 0.004), and group D versus group A 4.7 (95% CI, 2.12–10.4, p < 0.001).

## Discussion

In this study, we assessed interactions between MCV and RDW in predicting mortality among patients with stage 3–5 CKD, and found that the combination of both parameters improved the risk prediction for clinical outcomes, including all-cause mortality, and cardiovascular disease and infection-related mortality.

With respect to all-cause mortality using group A as the reference, the adjusted HRs were 1.44 and 2.14 for group B (higher RDW) and C (higher MCV), respectively. Mathematically, the adjusted HR for group D (both higher level of RDW and MCV) was expected to be 3.58 by adding 1.44 to 2.14. Actually, the adjusted HR was 5.06, which was higher than the expected value by the simple addition. Therefore, there was a multiplicative interaction between MCV and RDW in predicting overall mortality for patients with stage 3–5 CKD. The influences of RDW and MCV on the survival of the CKD patients were independent in the presence of other powerful predictors such as hemoglobin, albumin, renal function status and mineral disorders. Concerning the cause-specific mortality, there was also a trend of increasing mortality of CVD and infection from group A to D (P for trend <0.001 for both). Thus, patients with higher MCV and RDW levels were associated with the greatest risk of negative clinical outcomes.

RDW is an automatically calculated index in routine complete blood cell count reports. It is a numerical index that reflects variations in the size of red blood cells, and it is calculated by the following equation: (standard deviation of MCV ÷ mean MCV) × 100^15^. RDW has emerged as an independent prognostic factor in several medical conditions. However, little is known about the underlying mechanism by which an elevated level of RDW is associated with a negative impact on survival. RDW is commonly known to be elevated when there is an increase in the destruction of erythrocytes, and with increased and impaired erythropoiesis. An elevated RDW level has also been reported to reflect the presence of malnutrition, chronic inflammation and bone marrow dysfunction^9,16,17^. In addition, the prognostic implication of RDW has been reported to be similar in critically ill patients with a low CRP level compared to those with a high CRP level^18^. Therefore, increased inflammation may not fully explain the relationship between RDW and unfavorable outcomes. Another possible explanation is oxidative stress. It has been shown that high oxidative stress can impair erythropoiesis, shorten erythrocyte half- life and affect deformities in red blood cells which may ultimately result in increased RDW due to a mixed population of small and large erythrocytes in the circulation (anisocytosis)^19,20^. The release of heterogeneous erythrocytes with poor oxygen-carrying capacity into the peripheral circulation has been reported to disrupt the micro-circulation with local tissue hypoxia^21^. Although further studies are required to elucidate this hypothesis, it seems to be one of the most plausible mechanisms responsible for the association between RDW and negative clinical outcomes.

Erythrocytes are non-nucleated blood cells typically with an oval biconcave shape. The typical trait of anemia related to CKD is normocytic and normochromic in nature. In the general population, the causes of macrocytosis include vitamin B12 or folic acid deficiency, alcohol abuse, HIV medications, chemotherapeutics, reticulocytosis due to hemolysis or hemorrhage, hypothyroidism or liver disease^22^. Furthermore, bone marrow dysfunction has been reported to be a more common reason for macrocytic anemia in older patients than in younger patients^22^. The majority of clinical investigations on MCV in the prediction of mortality have focused on patients with malignancy. For example, the development of macrocytosis after capecitabine treatment has been shown to be a prognostic factor for a clinical response and overall survival in patients with advanced gastric cancer and breast cancer^23,24^. In addition, an elevation in MCV may be the result of impaired DNA biosynthesis caused by capecitabine-induced thymidylate synthetase inhibition^25,26^. Wenzel *et al*. demonstrated that a higher MCV value was more commonly noted in patients with a complete or partial response than in those with tumor progression^27^. Zheng *et al*. also concluded that a high preoperative MCV value was a negative prognostic predictor in esophageal squamous cell carcinoma independently of other risk factors^28^.

The clinical implications of MCV have also been addressed in patients with cardiovascular disease. For example, it has been shown to be positively correlated with atrial fibrillation (AF)^29,30^, and also to be significantly elevated in patients with chronic AF both before and after onset, suggesting that larger erythrocytes are already present before AF develops^29^. Likewise, MCV has been reported to be a potential risk factor for peripheral artery disease and to be associated with the severity^31,32^. Furthermore, Hatamian *et al*. studied 98 patients with ischemic stroke in middle cerebral artery territory and observed that MCV was a significant predictor for 1-week and 1-month mortality after ischemic stroke^33^. In addition, macrocytosis has been reported to be a predictor of survival in patients undergoing chronic hemodialysis^13^. Interestingly, a recent prospective study of 309 CKD patients showed that MCV was correlated with endothelial dysfunction and that it was an independent predictor for composite cardiovascular events^14^.

The mechanism responsible for the association between MCV and mortality is unknown, although several hypotheses have been proposed. One hypothesis involves oxidative stress, in that it has been implicated in a variety of chronic diseases, and the total antioxidant capacity of the body has been reported to be closely related to the size of circulating red blood cells^34^. As an elevated MCV level may reflect functional or structural disorders of the erythrocytes, the unfavorable impact of macrocytosis on survival can be explained by a deranged antioxidant capacity. In addition, impaired deformability of erythrocytes owing to high oxidative stress can further damage the microcirculation and oxygen delivery to tissues^35,36^. A second hypothesis is that macrocytosis may be a manifestation of deranged hematopoiesis due to bone marrow dysfunction. Bone marrow- derived mesenchymal stem cells have been reported to play a critical role in the repair of many damaged vital organs^37^. Another hypothesis is that nutrition may in part explain the association between MCV and patient survival. Crystal osmotic pressure, mainly determined by the serum concentrations of glucose, amino acids and electrolytes, is a key regulator of erythrocyte size^38^. Therefore, it is reasonable to speculate that malnourished patients are prone to higher MCV levels as a result of reduced serum osmotic pressure.

There are several potential limitations to this study associated its retrospective nature. First, we only took single measurements of MCV and RDW as the predictors for analysis, and thus the effect of variations in laboratory data over time was not evaluated which may have led to under-estimation of the association. Second, we did not assess the factors that can cause increased levels of RDW and MCV, such as acute bleeding (reticulocytosis), depletion of vitamin B12 or folic acid, and iron status. Because of the lack of laboratory data on blood levels of folic acid, vitamin B 12 and iron profile, the statistical analyses were instead adjusted for the use with folic acid, iron or vitamin B12. Although the utilization of certain medication does not guarantee appropriate replacement, some authors adopted the similar approaches. For example, the dietary intake of folate, vitamin B12 and iron was recorded for adjustments, rather than their respective serum levels^39^. Medication use of erythropoietin-stimulating agents, iron, folate and multivitamin was adjusted by others authors^13,17^. Furthermore, the study cohort was selected from the CKD care program in an outpatient setting, and was therefore relatively stable without acute events at the time of enrollment. Third, the lack of CRP for adjustment might bias our results. However, WBC was adjusted as a parameter for systemic inflammation. Finally, the most common cause of mortality was infection, followed by cardiovascular events in our study cohort. The discrepancy between ours and other studies might be attributable to the racial differences, diverse characteristics of study population or delivered healthcare. Nevertheless, the association between the interaction of MCV and RDW and overall mortality remained to be significant, irrespective of cause-specific mortality.

Consistent to our results, the multiplicative effect was also observed by a recent study of a large outpatient elderly population showing that the addition of MCV appears to improve the prognostic value of RDW as a predictor of overall survival^40^. Although the mechanism for our findings is unclear, the concurrent use of RDW and MCV may improve healthcare by identifying those at a greatest risk for mortality compared with the use of either RDW or MCV alone. The determination of MCV and RDW is not associated with any additional cost, and such data are widely available in complete blood cell count reports generated by hematological analyzers, whereas other novel prognostic parameters may be expensive and limited in a clinical setting. Therefore, we suggest that RDW and MCV may be novel risk factors that can be used in everyday clinical practice and should be considered to be additive parameters in risk stratification models for patients with CKD. Further large-scale prospective multi-center studies are required to validate our findings and elucidate the pathophysiologic mechanisms.

### Patients and methods

This is a retrospective cohort study conducted at a single medical center including patients with moderate to severe CKD. All of the enrolled subjects were diagnosed with CKD based on the National Kidney Foundation K/DOQI guidelines. The severity of CKD was defined by CKD stage according to estimated glomerular filtration rate (eGFR) calculated using the simplified 4-variable Modification of Diet in Renal Disease (MDRD) Study equation. Most of patients participating in the integrated CKD care program at our nephrology outpatient clinic were referred from primary care physicians or non-nephrology specialists for suspected or manifest renal failure. Enrollment criteria included patients with stage 3–5 CKD of at least 20 years old, less than 80 years, and with a follow-up period of at least 3 months. The final study cohort included 1075 patients with stage 3–5 CKD from January 1, 2006 to December 31, 2011. All of the patients were followed up from the index date, defined as the date when they joined the program, until death or the end of 2012. The Institutional Review Board of Changhua Christian Hospital approved the study protocol, and the investigation was carried out in compliance with the declaration of Helsinki. It is not necessary to obtain informed consent from study participants for a retrospective study in Taiwan. All of the data relevant to the patients were anonymized and de-identified before analysis.

Baseline variables at enrollment for each patient were obtained from medical records and a computerized electronic database, and included demographic data, medical history, body mass index (BMI), the underlying etiology of CKD, medical history, medication use, smoking and alcohol status, as well as laboratory data. The medical history consisted of diabetes mellitus (DM), hypertension, hyperlipidemia, coronary artery disease, congestive heart failure, cerebrovascular disease and peripheral artery disease, cancer, dementia, chronic lung disease, and liver cirrhosis. The history of medication use included angiotensin-converting enzyme (ACE) inhibitors, angiotensin II receptor blockers (ARB), anti-anemia agents (iron preparation, folic acid, and vitamin B12), erythropoiesis stimulating agents (ESA), and vitamin D. The laboratory tests included blood levels of blood urea nitrogen (BUN), creatinine, albumin, white blood cell (WBC) counts, hemoglobin, RDW, MCV, cholesterol, triglyceride, glutamic-pyruvic transaminase (GPT), uric acid, calcium, phosphate, and 24-hour proteinuria. Smoking habit was recorded as current or non-current smokers. Alcohol consumption was classified as never, former or current drinkers.

Complete blood cell counts were measured using an automatic hematology analyzer (DxH 800, Beckman Coulter). The upper normal limit of RDW is 14.9% at our laboratory. The optimal MCV cutoff value was determined to be 91.6 fL in receiver operator characteristic curve (ROC curve) analysis to best predict patient mortality, with a sensitivity of 0.627, specificity of 0.641 and area under the curve of 0.634 (95% CI, 0.587–0.681, p < 0.001). In order to assess the influence of the interaction between RDW and MCV on patient survival, the study cohort was categorized into four groups: group A (n = 415), RDW ≤ 14.9% and MCV ≤ 91.6 fL; group B (n = 232), RDW > 14.9% and MCV ≤ 91.6 fL; group C (n = 307), RDW ≤ 14.9% and MCV > 91.6 fL; and group D (n = 121), RDW > 14.9% and MCV > 91.6 fL. Cardiovascular disease (CVD) and infection were the two leading causes of death. Three outcomes were assessed: all-cause mortality, CVD-related mortality, and infection-related mortality.

### Statistical analysis

The whole study population was divided into four groups based on the optimal MCV value and the upper normal limit of RDW to facilitate the statistical analysis. The baseline characteristics of the study cohort stratified by MCV and RDW were expressed as mean ± standard deviation (SD) for numerical variables or number with percentage for categorical data. Comparisons of differences in baseline characteristics between the four groups were assessed using the chi-square test or Fisher’s exact test for categorical data, as appropriate. Analysis of variance (ANOVA) or the Kruskal-Wallis test was performed to assess differences in categorical covariates among the four groups.

For comparisons of survival status in the four groups, Kaplan-Meier survival curves were drawn and log-rank tests were performed to determine the statistical significance. Prognostic covariates for clinical outcomes were assessed using Cox proportional hazards models. An adjusted multivariate Cox proportional hazards model was used to determine the independent variables. The adjusted covariates included age, gender, BMI, smoking status, alcohol status, the causes of CKD, laboratory parameters, medication use, and comorbid conditions. The results of Cox regression analysis were expressed as hazard ratios (HR) and 95% confidence intervals (CI). All statistical analyses were carried out using IBM SPSS Statistics for Windows, Version 22.0 (IBM Corp., Armonk, NY). The results were considered to be statistically significant at a two-tailed p value < 0.05.
